# Effects of groundwater and distilled water on the durability of evaporitic rocks

**DOI:** 10.1038/s41598-023-32836-6

**Published:** 2023-04-06

**Authors:** Hasan Arman, Osman Abdelghany, Bahaa Mahmoud, Ala Aldahan, Safwan Paramban, Ahmed Gad, Mahmoud Abu Saima

**Affiliations:** 1 Geosciences Department, College of Science, United Arab Emirates University, P. O. Box: 15551, Al Ain, UAE; 2 Geology Department, Faculty of Science, Ain Shams University, Cairo, 11566 Egypt

**Keywords:** Environmental sciences, Natural hazards

## Abstract

Evaporitic rock durability induced by groundwater cause several construction challenges, but representative experimental studies to evaluate such conditions are still missing. Therefore, this study intended to provide better and more realistic degradability features of evaporites with groundwater besides a comparison with distilled water as slaking fluids. Forty-eight evaporitic rock blocks were collected from Abu Dhabi area, United Arab Emirates. 96 slake durability index (SDI) tests were performed, 48 with each of the slaking fluids; groundwater and distilled water, and their textural, mineralogical, and geochemical attributes were also examined before and after the SDI tests. In comparison to mineralogical and textural modifications, slaking fluid had a greater impact on the chemical composition of evaporitic rock. The study shows that the degradability of evaporites with groundwater and distilled water indicates a wide range from very low to high. The mean weight loss values after four cycles with groundwater and distilled water vary from 11 to 77 and from 4 to 81 wt.%, respectively. Consequently, slaking with groundwater illustrates a wide range compared to the slaking with distilled water. This could be due to quick interactions between groundwater and evaporites and fast hydration-dehydration process than distilled water due to the chemical composition of the groundwater. It is recommended to investigate the attributes of evaporitic rocks as well as groundwater geochemistry for safe, cost-effective, and sustainable structures.

## Introduction

Slake durability index (SDI) test is practical, has a relatively easy test sample preparation and is extensively used and accepted for assessing the durability of specifically soft, fragile and easily soluble rocks like evaporites, mudstone and shale. The SDI test is required for sustainable, durable, reliable and safe engineering structures and provides a critical index parameter (I_d_) for estimating the mechanical and chemical breakdown characteristics of rocks under cyclic (I_d1_–I_d4_) drying and wetting conditions with various slaking fluids. The mineralogical and textural features of rocks and the slaking fluid properties greatly control the rock degradability^1–16^.

The study of evaporitic rocks is valuable in basic geology, mineral deposits, petroleum geology, and engineering geology of which its dissolution has been a research highlight for several decades^17–21^. Subsurface evaporitic rocks are susceptible to being dissolved in water and forming cavities, sinkholes, and karst features, which can lead to unfortunate disasters due to the collapse of infrastructure, foundations, and dams^22–26^. So, it is critical to investigate the degradability of evaporitic rocks in order to provide significant long-term savings for mitigation measures and sustainable development.

Studies on evaporites (gypsum) degradability with different slaking fluids and the relation to their mineralogical and textural compositions are limited. Through field and laboratory studies, Yilmaz and Karacan^5^ investigated and correlated the slaking degradability and the textural features of various gypsum in terms of texture and crystal size. Their study revealed that fine-grained gypsum is more karstified and depicts a doline formation due to its crystal size, texture and effective porosity, which are essential parameters governing the gypsum slake durability and the doline formation. Meanwhile, Kayabali et al.^6^ performed many SDI tests on eight different gypsum types using various slaking fluids of the pH level to study the effects of mechanical and chemical slaking processes on the degradability of gypsum aside from the macroscopic and microscopic descriptions and XRD (X-ray diffraction) analyses of gypsum. They revealed that gypsum degradability ranges from medium to very high under various pH levels and the effect of the slaking fluid pH levels is limited in the slaking durability of gypsum. By contrast, the slake durability of gypsum is considerably affected by its mineralogical and textural composition. Rahimi et al.^11^ studied the mineral composition and texture effects on the durability of sulfate rocks, they applied slake durability test in 5 cycles of 10 min using ionized water under dry and saturated conditions, these tests have revealed that these sulfate rock contain gypsum and anhydrite and a rock's mineral composition affects how durable it is. Their study showed that in both dry and saturated conditions, the durability index drops as the gypsum percentage rises, and inversely, it rises when the anhydrite content rises. Their study proved that rock texture has a significant impact on the durability index that’s why in a same mineral composition, a rock with a porphyry texture is more durable than a rock with an alabaster texture.

It is critical to investigate some other engineering characteristics with respect to the slake durability test. Arman^12^ predicted the relations between uniaxial compressive strength (UCS) and indirect tensile strength (Brazilian) (ITS) and the UCS and 2nd cycle of slake durability index (I_d2_) of 48 representative evaporitic rock samples with characteristic empirical equations. Even though the correlation between them was moderate, the Student’s t and F tests proved that the correlation between the UCS and the ITS and the I_d2_ was significant. His study exposed that the proposed empirical relationship was appropriate and the UCS of evaporitic rocks within a certain correlation coefficient (R) could be foreseen using the ITS and the I_d2_. Arman et al.^13,14^ studied the slaking characteristics of evaporitic rocks with the combination of the mineralogical and textural examinations of representative test samples before and after slaking tests. The slake test data showed that the evaporite durability ranged from high to very low and medium to very low. The average weight loss value after four successive cycles (I_d1_–I_d4_) were 40–93 and 24–95 wt.%, which indicated a fast development of the hydration–dehydration effects due to the chemical and mineralogical compositions of evaporites. Moreover, Arman et al.^15^ investigated the petrographical and geoengineering characteristics of the evaporitic rocks in the city of Abu Dhabi, they performed all the rock mechanical tests including slake durability test to get the geoengineering properties such as strength and durability, also they applied SEM, XRD, X-ray fluorescence (XRF) analyses to obtain the petrographical features. They found that relationship between geoengineering features exit the various degrees of correlation such as weak to strong, positive to negative possibly highlight the effect of petrographical features of the evaporitic rocks on these parameters.

The present study aims to investigate the effects of groundwater and distilled water as the slaking fluids on the evaporite degradability after four consecutively cycles (I_d1_–I_d4_), evaluate and discuss why using groundwater as a slaking fluid is better and realistic and compare the SDI results in the case of using groundwater and distilled water as the slaking fluids. The mineralogical and textural compositions of the evaporite samples used for the SDI tests are examined before and after each test to appraise their possible influences on the evaporite durability. These combined studies may help engineers alleviate the durability problems associated with the evaporites in the study area and some other similar areas.

## Field and laboratory studies

**Figure 1:**
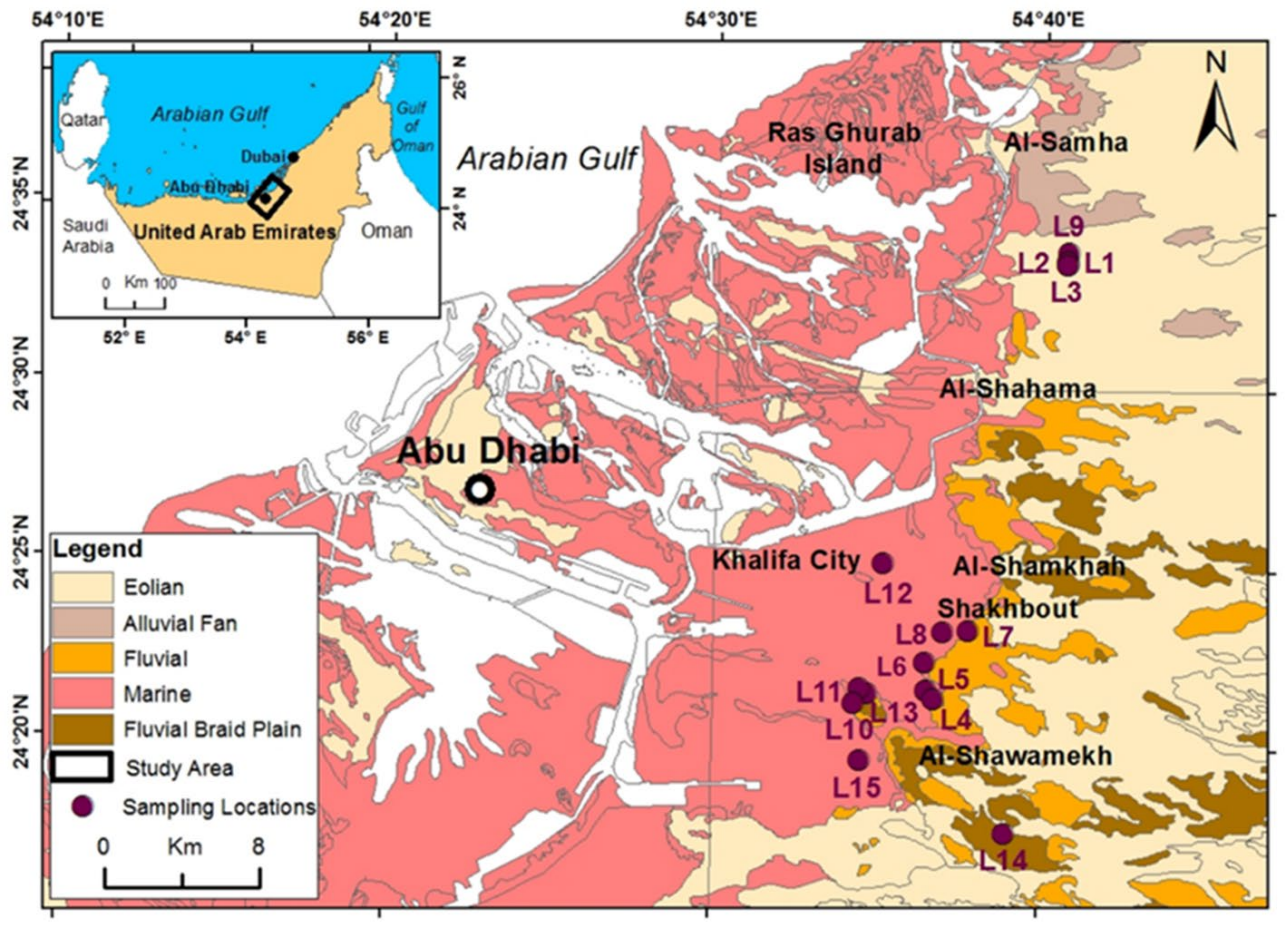
Geological map of the study area and sampling locations (generated with ArcGIS 10.8^27^).

**Figure 2:**
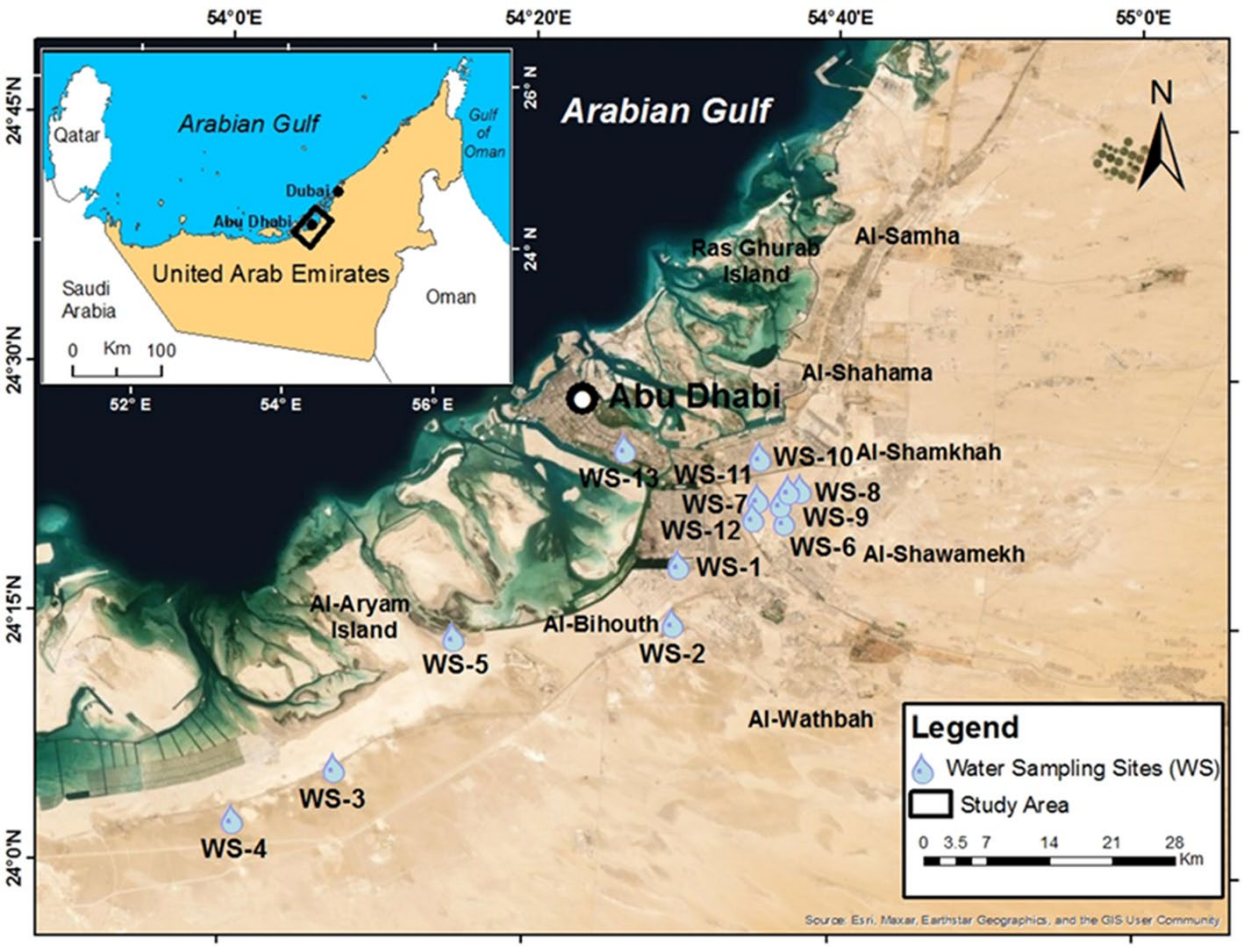
Groundwater sampling sites of the study area and surroundings areas (generated with ArcGIS 10.8^27^).

The study area is Abu Dhabi and its surrounding areas. The general land surface of the study area from top to bottom is approximately 2 m of dune sand, 1–2-m-thick gypcrete soil resultant from recent aeolian, an evaporite sequence of gypsiferous layers interbedded with mudstone and Lower Miocene-age evaporitic rocks approximately 18–20 m deep (Fig. 1). Evaporitic rock blocks (48) and groundwater samples (approximately 200 L in 20 L plastic bottles) were collected from 15 (Fig. 1) and 13 (Fig. 2) locations in the study area, respectively.

The studied evaporitic rock block samples were classified into groups 1–4 according to the petrographic description^28^ of their fresh samples and the thin-section images under plane and cross-polarized microscopes (Nikon, LV 100N POL with NIS Elements imaging software^29^). Four variations in the mineralogical and textural morphologies were mainly related to the crystallization process of the evaporitic rocks and the hydration–dehydration conditions.

**Figure 3:**
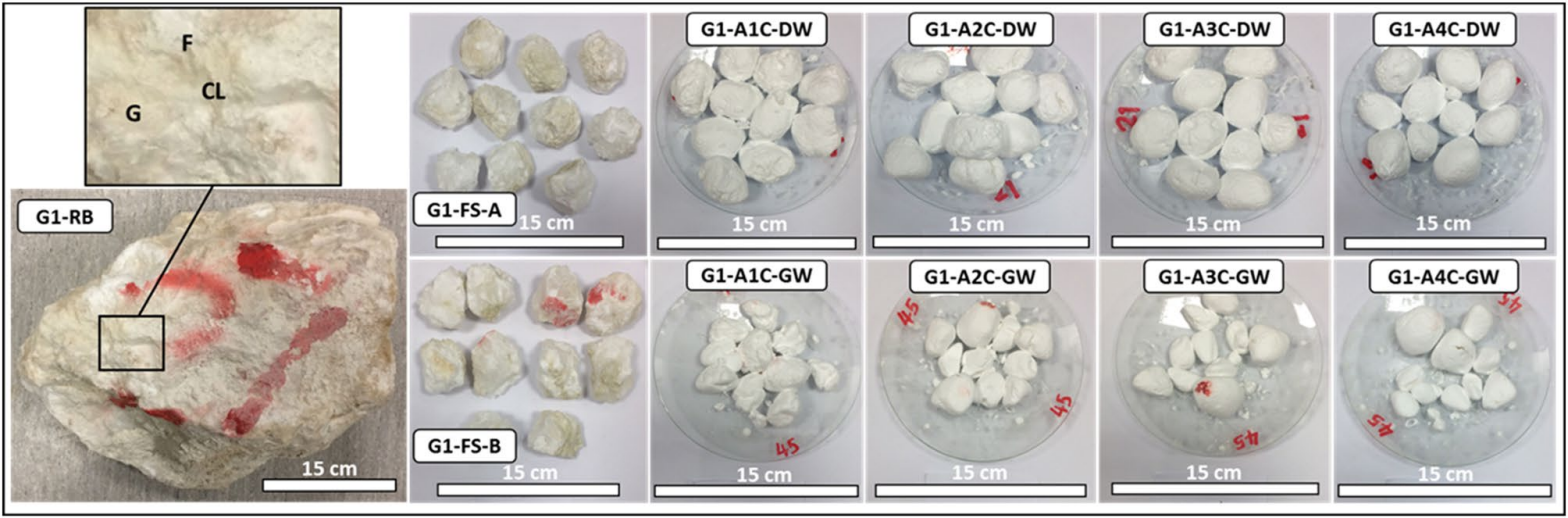
Representative evaporitic rock block (G1–RB; Group 1, Rock Block) and slake durability test samples cycled with distilled water and groundwater (1st–4th cycles). G1-FS-A represents Group 1, fresh sample, sample A, G1-A1C-DW to G1-A4C-DW after 1st–4th cycles with distilled water. G1-FS-B represents Group 1, fresh sample, sample B, G1-A1C-GW to G1-A4C-GW after 1st–4th cycles with groundwater. G = Gypsum, F = Fracture, CL = Clay.

**Figure 4:**
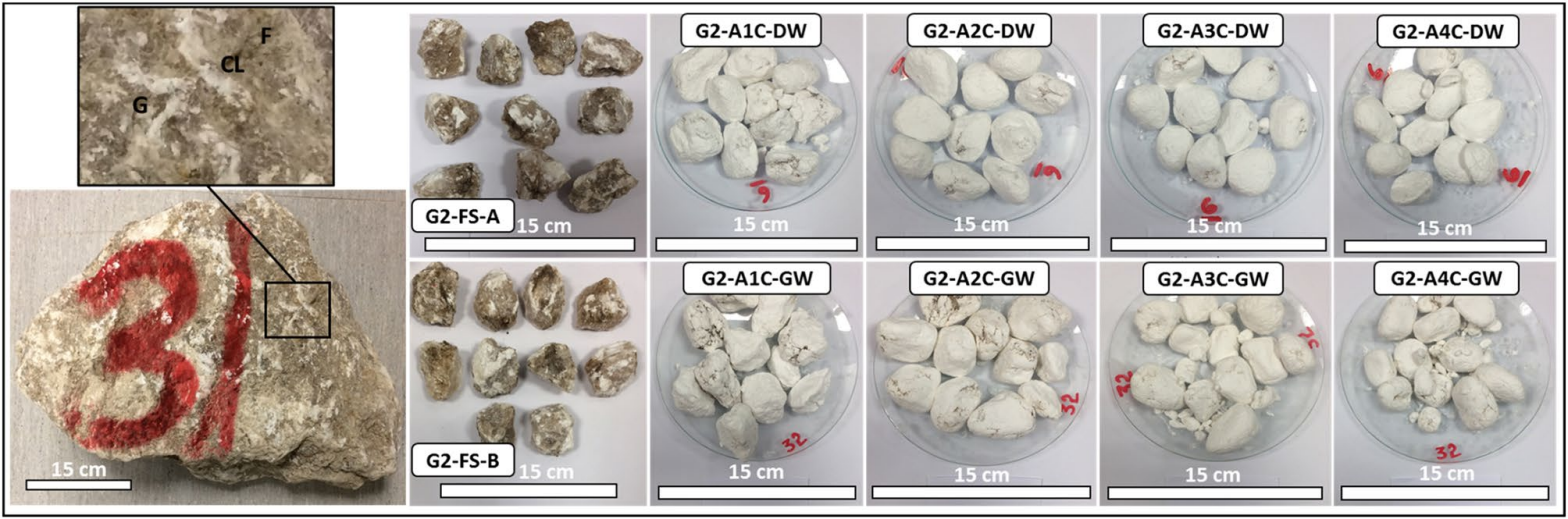
Representative evaporitic rock block (G2–RB; Group 2, Rock Block) and slake durability test samples cycled with distilled water and groundwater (1st–4th cycles). G2-FS-A represents Group 2, fresh sample, sample A, G2-A1C-DW to G2-A4C-DW after 1st–4th cycles with distilled water. G2-FS-B represents Group 2, fresh sample, sample B, G2-A1C-GW to G2-A4C-GW after 1st–4th cycles with groundwater. G = Gypsum, F = Fracture, CL = Clay.

**Figure 5:**
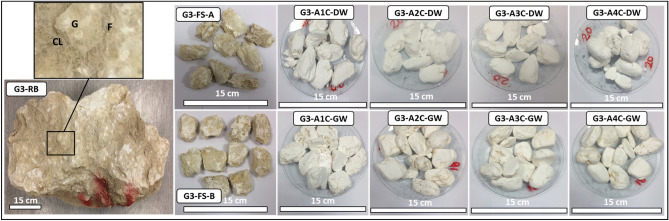
Representative evaporitic rock block (G3–RB; Group 3, Rock Block) and slake durability test samples cycled with distilled water and groundwater (1st–4th cycles). G3-FS-A represents Group 3, fresh sample, sample A, G3-A1C-DW to G3-A4C-DW after 1st–4th cycles with distilled water. G3-FS-B represents Group 3, fresh sample, sample B, G3-A1C-GW to G3-A4C-GW after 1st–4th cycles with groundwater. G = Gypsum, F = Fracture, CL = Clay.

**Figure 6:**
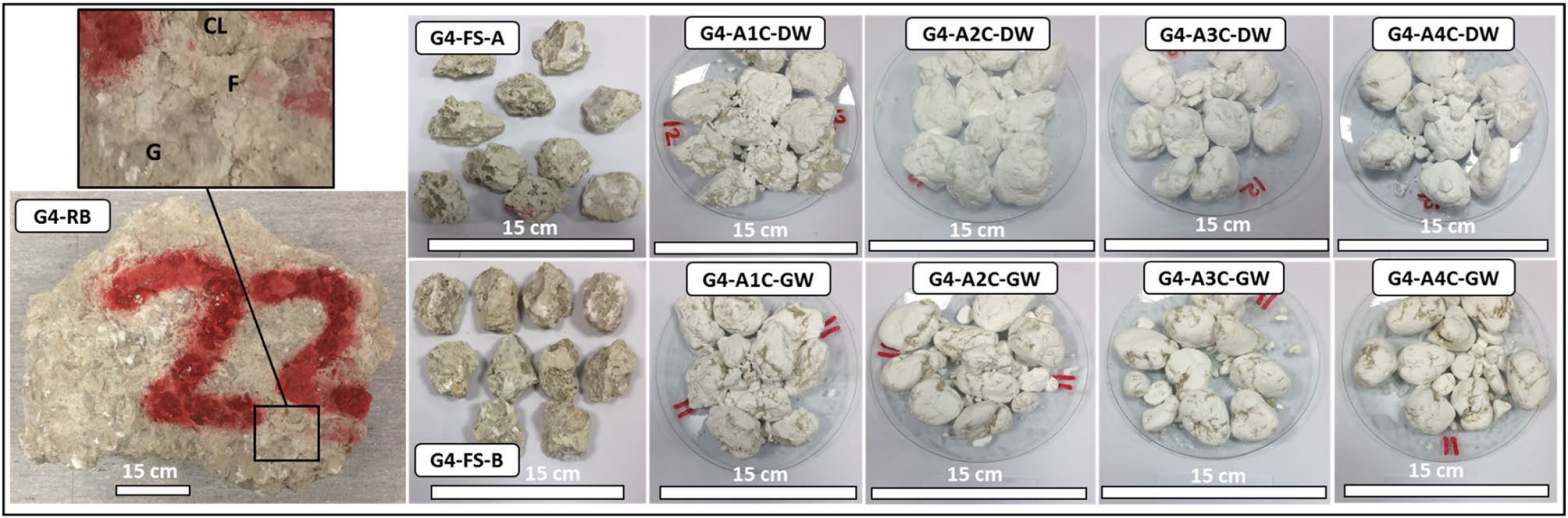
Representative evaporitic rock block (G4–RB; Group 4, Rock Block) and slake durability test samples cycled with distilled water and groundwater (1st–4th cycles). G1-FS-A represents Group 4, fresh sample, sample A, G4-A1C-DW to G4-A4C-DW after 1st–4th cycles with distilled water. G4-FS-B represents Group 4, fresh sample, sample B, G4-A1C-GW to G4-A4C-GW after 1st–4th cycles with groundwater. G = Gypsum, F = Fracture, CL = Clay.

Forty-eight representative SDI test samples, with each test set including 10 rock lumps within 40–60 g each, were prepared. With groundwater and distilled water as the slaking fluids, the SDI tests were performed according to the American Society for Testing and Materials^30^. Figures 3, 4, 5, 6 show the representative evaporitic rock block for each group (e.g., G1–RB; Group 1, Rock Block), prepared fresh SDI test samples A and B (e.g., G1–FS–A and G1–FS–B; Group 1, Fresh Sample, samples A and B) and samples A and B after one to four cycles with distilled water and groundwater (e.g., G1–A1C–4C–DW and G1–B1C–4C–GW; Group 1, after one to four cycles, distilled water and groundwater).

The mineralogical composition and the textural features of the evaporitic rock samples collected from different locations and representative of the four groups (Table 1, e.g., L15–37–1; Location 15, Rock block number 37, Sample number 1) were studied using a cross-polarized light microscope and SEM (FEI INCA PentaFETX3 with high vacuum, electron beam voltage 200 V–30 kV, and imaging using BSE detector), XRD (Compact Aeris with Empyrean X-ray tube Co LFF and the highly acclaimed PIXcel1D line detector), and XRF (Epsilon 1– EDXRF with SDD10P, MCA TYPE: PAN-DPP-Compact Detector and Ag50 Tube, Kv: 7.000–50.000, Max. µA: 1000, Max. Watts: 10.000) analyses performed on the powdered sample aliquots before and after the slaking tests. Samples preparation, petrographic examinations, SEM, XRD, XRF, and SDI analyses were conducted at the Geosciences Department, College of Science, United Arab Emirates University. To ensure the analytical performance of the Compact Aeris and Epsilon 1– EDXRF, all XRD and XRF operating parameters were software controlled^31,32^. They were calibrated using standard reference materials 45W9202-Calcite, A1070-3590A5, and Cu-3590C2. All reagents used were analytical grade and all solutions were prepared using double distilled water.

**Table 1:** Chemical composition of representative evaporitic samples from group 1 to 4 before and after slaking with distilled water and groundwater.

Sample ID#	Slaking fluid	wt. (%)	CaO	SO_3_	Na_2_O	MgO	Al_2_O_3_	SiO_2_	P_2_O_5_	K_2_O	Fe_2_O_3_	TiO_2_	Ti	Cl	Mn	Sr	Ba	Zr
Group 1 (L15—37–1)		Before slaking	47.644	51.275	0.000	0.510	0.112	0.155	0.014	0.000	0.013	0.003	0.002	0.139	0.000	0.087	0.021	0.001
	Distilled water	After slaking	46.876	52.165	0000	0.577	0.106	0.019	0.060	0.000	0.003	0.000	0.000	0.031	0.000	0.130	0.004	0.001
	Groundwater*	After slaking	44.418	49.807	0.623	0.887	0.089	0.039	0.000	0.116	0.005	0.000	0.000	3.838	0.000	0.130	0.001	0.001
Group 2 (L11–31–1)		Before slaking	47.120	50.682	0.576	0.524	0.123	0.584	0.000	0.041	0.101	0.040	0.024	0.155	0.000	0.075	0.018	0.024
	Distilled water	After slaking	46.380	51.753	0.853	0.585	0.125	0.064	0.047	0.000	0.022	0.000	0.000	0.031	0.000	0.094	0.017	0.001
	Groundwater*	After slaking	43.661	49.498	2.404	0.778	0.07	0.05	0.000	0.105	0.006	0.000	0.000	3.282	0.000	0.095	0.018	0.001
Group 3 (L5–23–8)		Before slaking	47.478	50.527	0.038	0.904	0.159	0.438	0.021	0.054	0.067	0.033	0.020	0.214	0.001	0.107	0.022	0.028
	Distilled water	After slaking	46.989	51.867	0.068	0.623	0.082	0.030	0.048	0.000	0.014	0.000	0.000	0.097	0.000	0.129	0.023	0.001
	Groundwater*	After slaking	46.773	48.599	0.000	0.835	0.080	0.051	0.031	0.164	0.011	0.000	0.000	3.281	0.000	0.113	0.016	0.001
Group 4 (L4–22–3)		Before slaking	48.514	44.660	0.000	2.008	0.342	2.342	0.011	0.208	0.400	0.075	0.045	1.329	0.005	0.111	0.033	0.051
	Distilled water	After slaking	45.802	50.911	1.265	0.839	0.093	0.352	0.000	0.062	0.082	0.005	0.003	0.369	0.001	0.175	0.012	0.002
	Groundwater*	After slaking	44.592	46.220	1.687	0.944	0.071	0.25	0.082	0.203	0.058	0.002	0.001	5.65	0.000	0.180	0.019	0.001
Total sample = 4																		

*Groundwater specification: Groundwater level = 10–40 m, Temperature = 21.5–31.2 ($^\circ$C), Salinity = 20,000–65,000 (ppm), pH = 7.14–9.45, TDS = 32,700–175,360 (ppm), EC = 28,100–274,000 (μ mhos/cm).

## Results and discussion

### Rock blocks and thin sections description

#### Group 1

**Figure 7:**
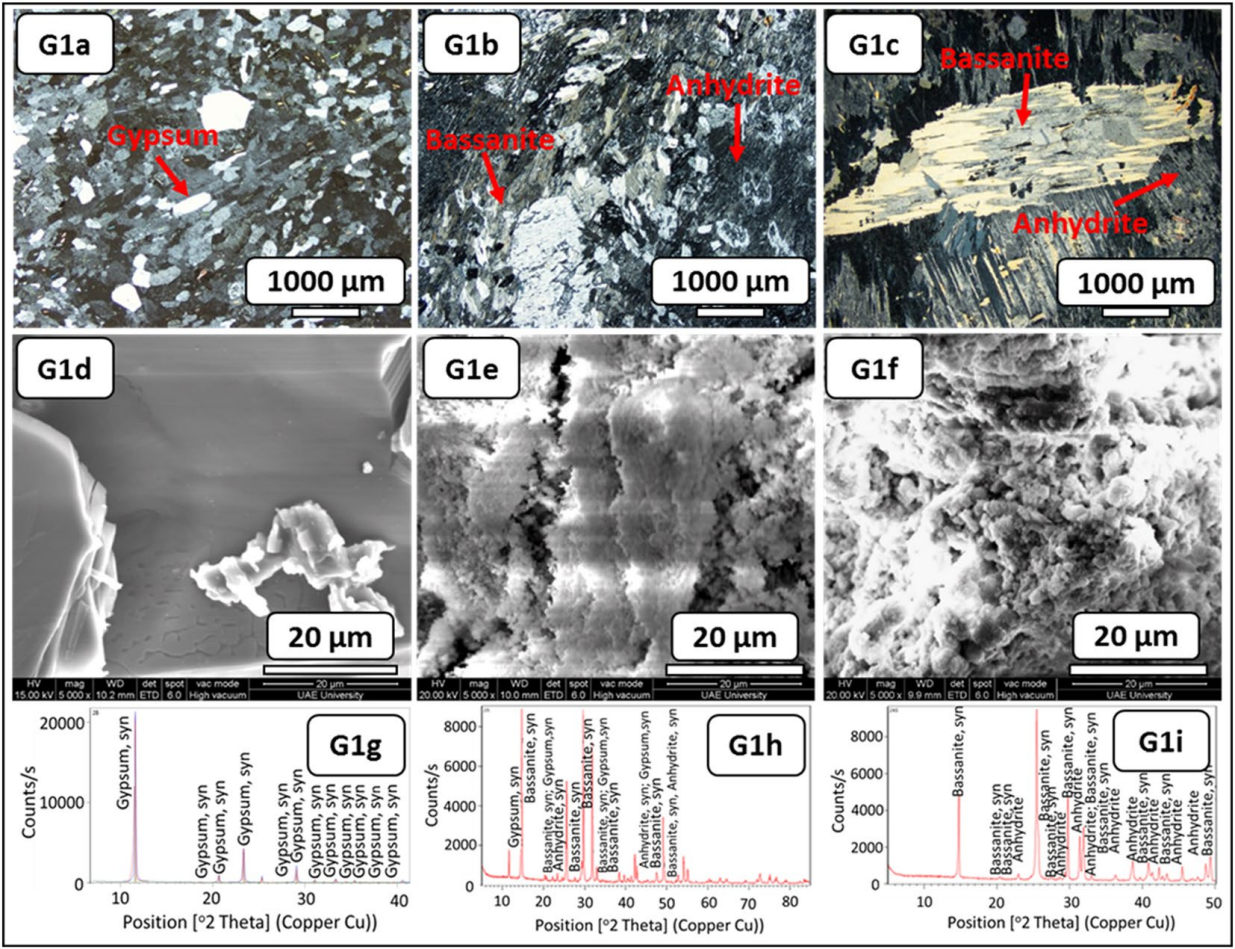
Microscopic description, SEM and XRD of an evaporitic rock Group 1 (fresh sample, after slaking with distilled water and groundwater respectively). G1a-G1c represents thin section images under cross-polarized microscope showing mega crystalline pure prismatic gypsum crystals before SDI and fibro-radiating basanite intergrowth with microcrystalline anhydrite after SDI test. G1d-G1f. represents SEM images showing tabular gypsum and needle shaped before SDI with interspersed in a loose fabric and filling anhydrite in the microfractures after SDI test. G1g-G1i represents XRD analyses showing dominant of gypsum before SDI test where after SDI dominance of hemihydrate- dehydrated calcium sulfate.

The examination of the fresh rock block samples indicated large gypsum crystals with a white to transparent color and minor fractures filled with mudstones (Fig. 3, G1–RB). The thin-section study before the SDI test showed mega to meso-crystalline prismatic gypsum in a porphyrobalstic texture. The gypsum crystals denoted a few anhydrite lath inclusions (Fig. 7, G1a). After the SDI test with distilled water, meso-crystalline-radiating and needle-like bassanite crystals were recognized, which were embedded in microcrystalline anhydrite groundmass and poorly clay patches (Fig. 7, G1b). Meso-crystalline fibro-radiating bassanite intergrowth with micro-crystalline anhydrite and bassanite crystals embedded in poorly micro-crystalline bassanite or anhydrite groundmass with clay patches were detected after the SDI tests with groundwater. Preserved outlines of prismatic gypsum crystals were also observed (Fig. 7, G1c).

#### Group 2

**Figure 8:**
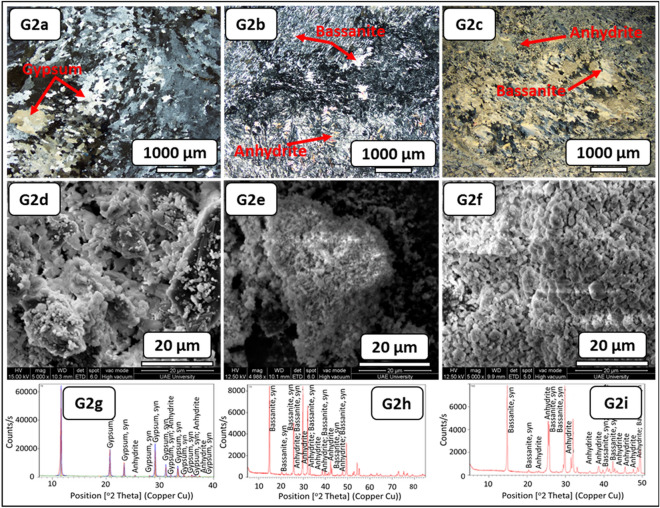
Microscopic description, SEM and XRD of an evaporitic rock Group 2 (fresh sample, after slaking with distilled water and groundwater respectively). G2a-G2c represents thin section images under cross-polarized microscope showing meso-crystalline gypsum with minor anhydrite crystals before SDI and alignment of microcrystalline fibrous bassanite and anhydrite crystals after SDI test. G2d-G2f. represents SEM images showing gypsum aggregates network before SDI changed to fibrous anhydrite, bassanite crystals and aggregates after SDI test. G2g-G2i represents XRD analyses showing dominant of gypsum with some anhydrite before SDI test whereas after SDI dominance of bassanite and anhydrite.

In Fig. 4, G2–RB, the fresh block samples exhibited gypsum with a reddish honey color, large crystals, and minor fractures filled with mudstones. The thin-section investigation before the SDI test displayed meso-crystalline to micro-crystalline irregular gypsum crystals. A few dispersed anhydrite crystals were confirmed (Fig. 8, G2a). Subsequently, the alignment of micro-crystalline fibrous bassanite crystals with few anhydrite laths intergrowth with micro-crystalline bassanite or anhydrite groundmass was observed after the SDI test using distilled water (Fig. 8, G2b). After the SDI test using groundwater as the slaking fluid, micro-crystalline parallel to the subparallel fibrous bassanite crystals embedded in the poorly defined micro-crystalline bassanite or anhydrite groundmass with few anhydrite laths was perceived. Some gypsum crystals were detected with aphanitic carbonate crystals (Fig. 8, G2c).

#### Group 3

**Figure 9:**
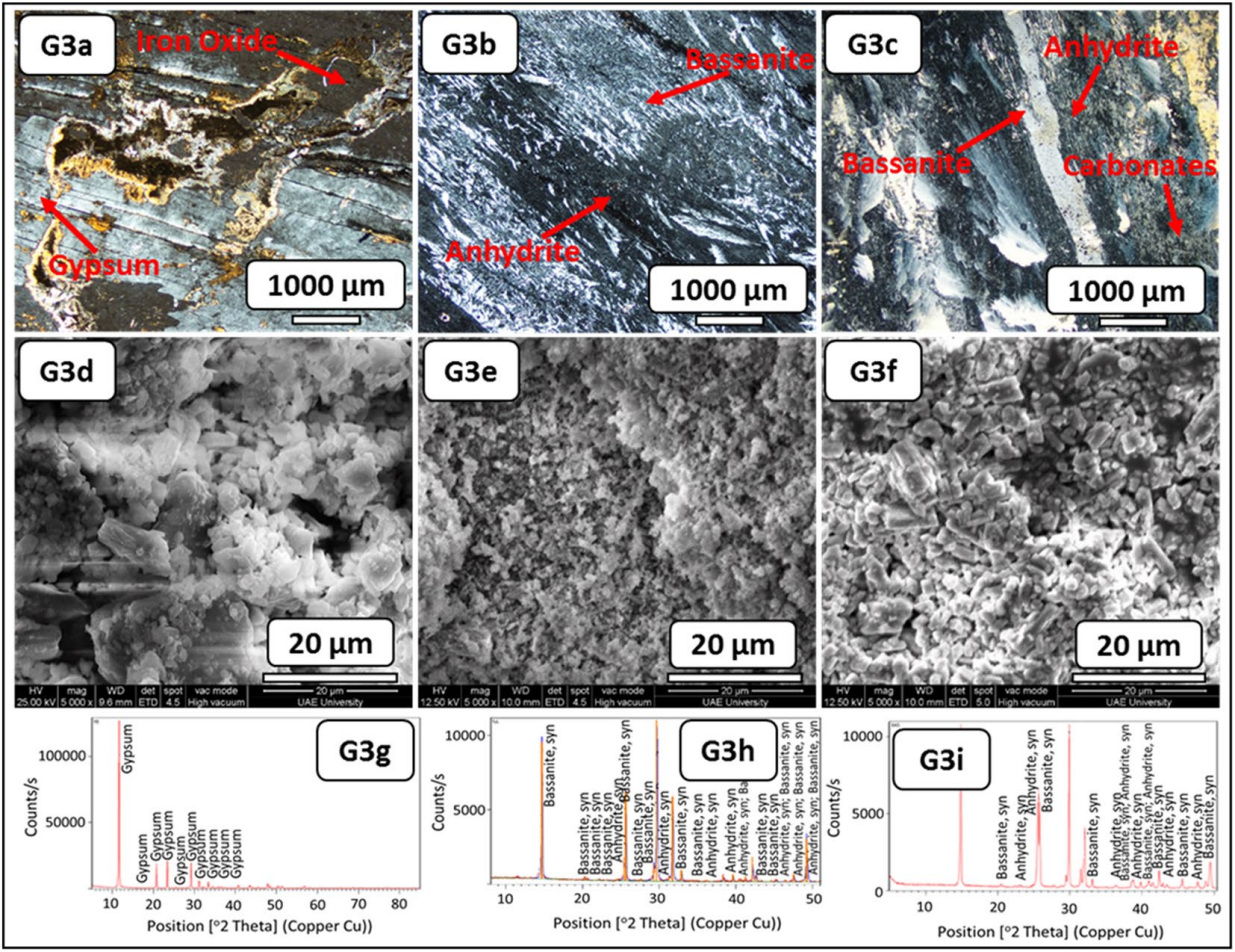
Microscopic description, SEM and XRD of an evaporitic rock Group 3 (fresh sample, after slaking with distilled water and groundwater respectively). G3a–G3c represents thin section images under cross-polarized microscope showing mega- bladed crystals of gypsum with iron oxides patches before SDI and fibro-radiating bassanite intergrowth with microcrystalline anhydrite with trace carbonate after SDI test. G3–-G3f. represents SEM images showing prismatic gypsum crystals and needle shaped before SDI with interspersed in a loose fabric of bassanite and anhydrite after SDI test. G3g-G3i represents XRD analyses showing richness of gypsum before SDI test where after SDI bassanite is mainly dominant than anhydrite.

The macro description of the fresh block samples displayed gypsum that was yellowish-white with anhydrite and large fractures filled with yellow mudstones (Fig. 5, G3–RB). The thin-section examination before the SDI test demonstrated mega crystalline interlocked bladed fractured gypsum with anhydrite crystals. This evaporitic rock was rich with clay patches, micro-crystalline carbonate, and iron oxides (Fig. 9, G3a). Micro-crystalline fibro-radiating bassanite crystal intergrowth with anhydrite was documented after the SDI test with distilled water. A few clay patches and aphanitic carbonate crystals were also observed (Fig. 9, G3b). After the SDI test with groundwater, meso-crystalline fibro-radiating bassanite crystals intergrowth with anhydrite were observed, which were very rich with randomly oriented anhydrite laths. Very few clay patches and aphanitic carbonate crystals were also noticed (Fig. 9, G3c).

#### Group 4

**Figure 10:**
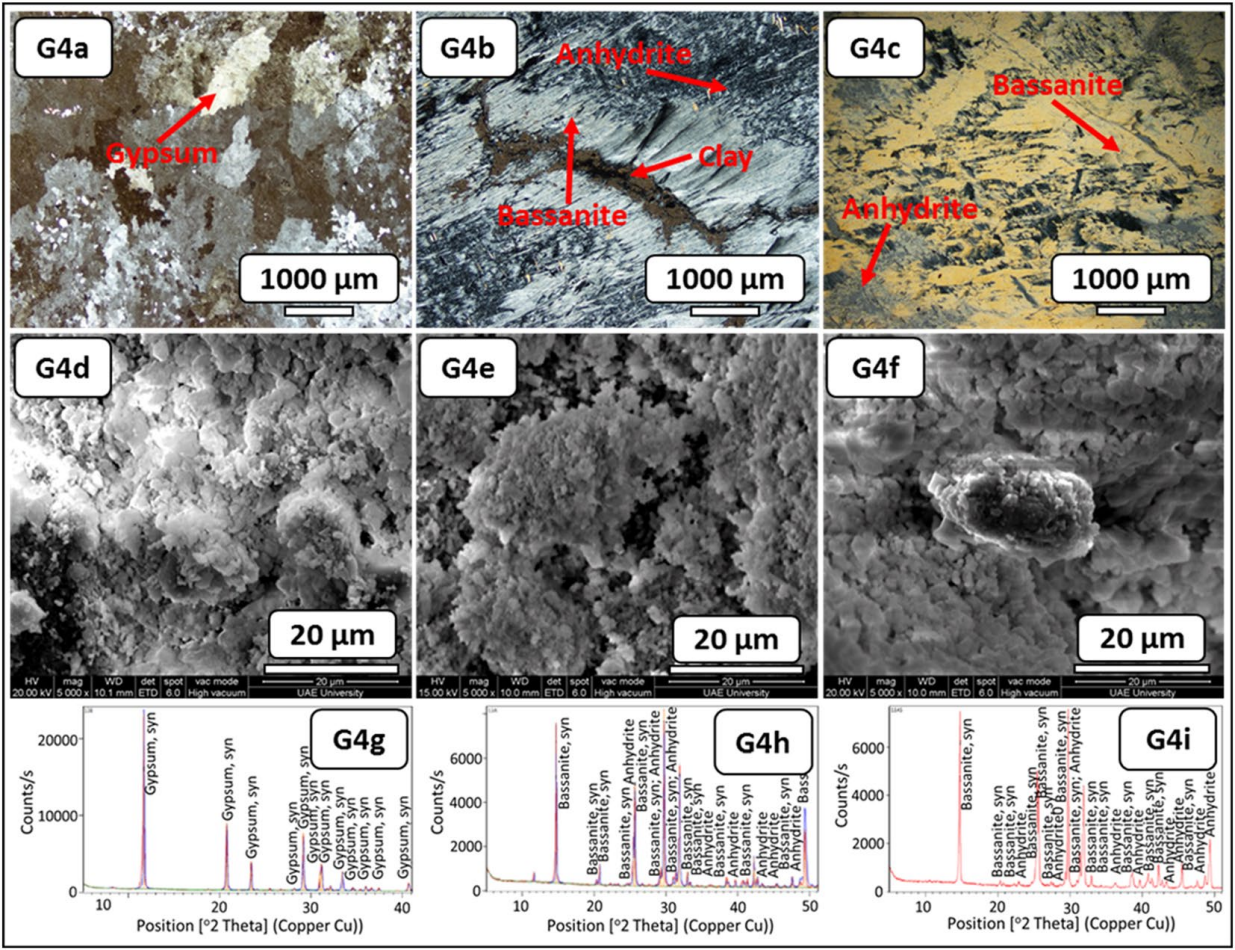
Microscopic description, SEM and XRD of an evaporitic rock Group 4 (fresh sample, after slaking with distilled water and groundwater respectively). G4a–G4c represents thin section images under cross-polarized microscope showing mega-crystalline irregular gypsum crystals intergrowth in mosaic crystals before SDI and fibro-radiating bassanite intergrowth with microcrystalline anhydrite rich with clay patches, after SDI test. G4d-G4f. represents SEM images showing gypsum aggregates before SDI with fibrous mixed bassanite and anhydrite crystals and aggregates after SDI test. G4g–G4i represents XRD analyses showing dominant of gypsum before SDI test where after SDI dominance of bassanite-anhydrite.

The visual inspection of the fresh block samples paraded gypsum with a greenish-grey color, nodular and fractures filled with green mudstones (Fig. 6, G4–RB). The thin-section study before the SDI test revealed a mega-crystalline irregular gypsum crystal intergrowth with meso-crystalline mosaic crystals. Some crystals showed anhydrite lath inclusions. This evaporitic rock was rich with clay patches and carbonate (Fig. 10, G4a). After the SDI tests using distilled water, traces of the gypsum remain were observed with the micro-crystalline fibro-radiating bassanite crystal intergrowth with fibrous anhydrite. It also had clay patches, some iron oxides, and carbonate (Fig. 10, G4b). After the SDI test using groundwater as the slaking fluid, gypsum crystals were replaced by meso-crystalline fibro-radiating bassanite and anhydrite crystals (Fig. 10, G4c).

### SEM, XRD and XRF analyses

The morphological changes during the course of hydration (slaking with fluids of distilled and groundwater) were investigated through the SEM technique (Figs. 6, 7, 8, 9, 10, G1e and G1f–G4e and G4f ). The morphology of the gypsum crystals before the SDI test was tabular in nature, needle-shaped, and rod-like. Raw gypsum with tiny particles (< 2 µm) was observed on the surface of larger particles (Figs. 6, 7, 8, 9, 10, G1d–G4d). After the SDI test with distilled water, the tiny particles disappeared, and the large particles were broken into smaller ones. The shape of the crystals was deformed, and their morphologies differed. Clusters of anhydrites were also present in-between the bassanite crystals (Figs. 7, 8, 9, 10, G1e–G4e). After the SDI test with groundwater, Figs. 7, 8, 9, 10, G1f–G4f showed the typical microstructure of hemihydrate- dehydrated calcium sulfate forming an open structure.

The XRD analysis interprets the positional relationship of diffracted intensities. Each crystalline phase shows a unique diffraction pattern for identifying different minerals. Particularly, anhydrite (CaSO_4_) can easily be distinguished by their XRD patterns (Figs. 7, 8, 9, 10, G1h–G1i–G4h–G4i). Depending on the amount of water within the crystal structure, calcium sulfates were found in four different phases: gypsum (CaSO_4_·2H_2_O), bassanite (CaSO_4_·0.5H_2_O), and anhydrite (CaSO_4_) and sulfate hannebachite (CaSO_3_·0.5H_2_O). The fresh samples from the studied areas were analyzed using XRD, which revealed that gypsum was dominant in the fresh samples in addition to calcite and halite (Figs. 7, 8, 9, 10, G1g–G4g), wherefore bassanite (CaSO_4_·0.5H_2_O) and anhydrite (CaSO_4_) were dominant after the SDI test with distilled water or groundwater (Figs. 7, 8, 9, 10, G1h and G1i–G4h and G4i ). The repeated drying of the samples in the oven at 12–16 h, 105 C° temperature^18^ led to the dehydration of gypsum to bassanite and anhydrite.

The chemical compositions of the different fresh gypsum samples were obtained via XRF and expressed as oxides wt.% in Table 1. CaO was averaged as 47.494 wt.%. The SO_3_ content was higher, averaging 50.074 wt.% (Table 2). Table 2 presents lower SiO_2_ (0.613 wt.%), MgO (0.844 wt.%), Al_2_O_3_ (0.164 wt.%) and Fe_2_O_3_ (0.122 wt.%) contents. The other trace elements of low values (e.g., Sr, Ba and Zr) indicated that the gypsum samples are of considerable purity However, after the SDI tests using distilled water (Table 2), the average CaO of 46.519 wt.% was lower, whereas the average SO_3_ of 51.835 wt.% was higher than for the fresh gypsum samples. The other oxides and trace elements remained low, showing a slight difference compared to the fresh gypsum samples. Tables 1 and 2 show the chemical analysis results for the samples after the SDI tests with the groundwater. In Table 2, the average of CaO 39.801 wt.% and SO_3_ 42.523 wt.% represented lower contents compared to those for the fresh samples or those tested with distilled water. The averages of the Na_2_O 0.673 wt.% and Cl 3.220% values were higher than those found for the fresh gypsum and distilled water SDI-tested samples. This was the result of the high NaCl content of the groundwater used in the SDI tests. Other oxides and trace elements had low concentrations and little variability (Tables 1 and 2). In conclusion, the fresh gypsum samples were not significantly chemically changed by the SDI using distilled water. However, significant changes in Na_2_O and Cl content were found when groundwater was used in the SDI tests as a slaking fluid. After SDI with different slaking fluids, the combined values of Al_2_O_3_, SiO_2_, Fe_2_O_3_, and TiO_2_ decreased, resulting in an increase in SO_3_%. These oxides are associated with sample impurities (clay minerals and iron oxides), which are easily removed by slaking fluids. In contrast, Na_2_O, K_2_O, and Cl values increased after SDI with groundwater (high salinity), implying that SO_3_ is reduced. The observed decrease in CaO content after SDI with different fluids may be attributed to the association of calcium ions in different mineral phases such as dolomite and clay minerals, which are typically present as mineral impurities in evaporite rocks.

**Table 2:** Average chemical composition variation of evaporitic samples before and after slaking with distilled water and groundwater.

	wt. (%)	CaO	SO_3_	Na_2_O	MgO	Al_2_O_3_	SiO_2_	P_2_O_5_	K_2_O	Fe_2_O_3_	TiO_2_	Ti	Cl	Mn	Sr	Ba	Zr
Slaking fluid	Before slaking	47.494	50.074	0.25	0.844	0.164	0.613	0.023	0.051	0.122	0.024	0.015	0.240	0.001	0.088	0.019	0.013
Distilled water	After slaking	46.519	51.835	0.466	0.643	0.108	0.106	0.036	0.014	0.036	0.001	0.001	0.078	0.000	0.114	0.015	0.001
	Differences	0.975	−1.761	−0.216	0.201	0.056	0.507	−0.013	0.037	0.086	0.023	0.014	0.161	0.001	−0.026	0.004	0.012
Groundwater*	After slaking	39.801	42.523	0.673	0.707	0.076	0.121	0.021	0.122	0.039	0.001	0.001	3.220	0.000	0.144	0.017	0.001
	Differences	7.693	7.755	−0.423	0.137	0.088	0.492	0.002	−0.071	0.083	0.023	0.014	−2.980	0.001	−0.056	0.002	0.012
Total samples = 14–16**																	

*Groundwater specification: Groundwater level = 10–40 m, Temperature = 21.5–31.2 ($^\circ$C), Salinity = 20,000–65,000 (ppm), pH = 7.14–9.45, TDS = 32,700–175,360 (ppm), EC = 28,100–274,000 (μ mhos/cm).

**Chemical composition of two samples were missing in case of groundwater as a slaking fluid.

### Slake durability tests

**Figure 11:**
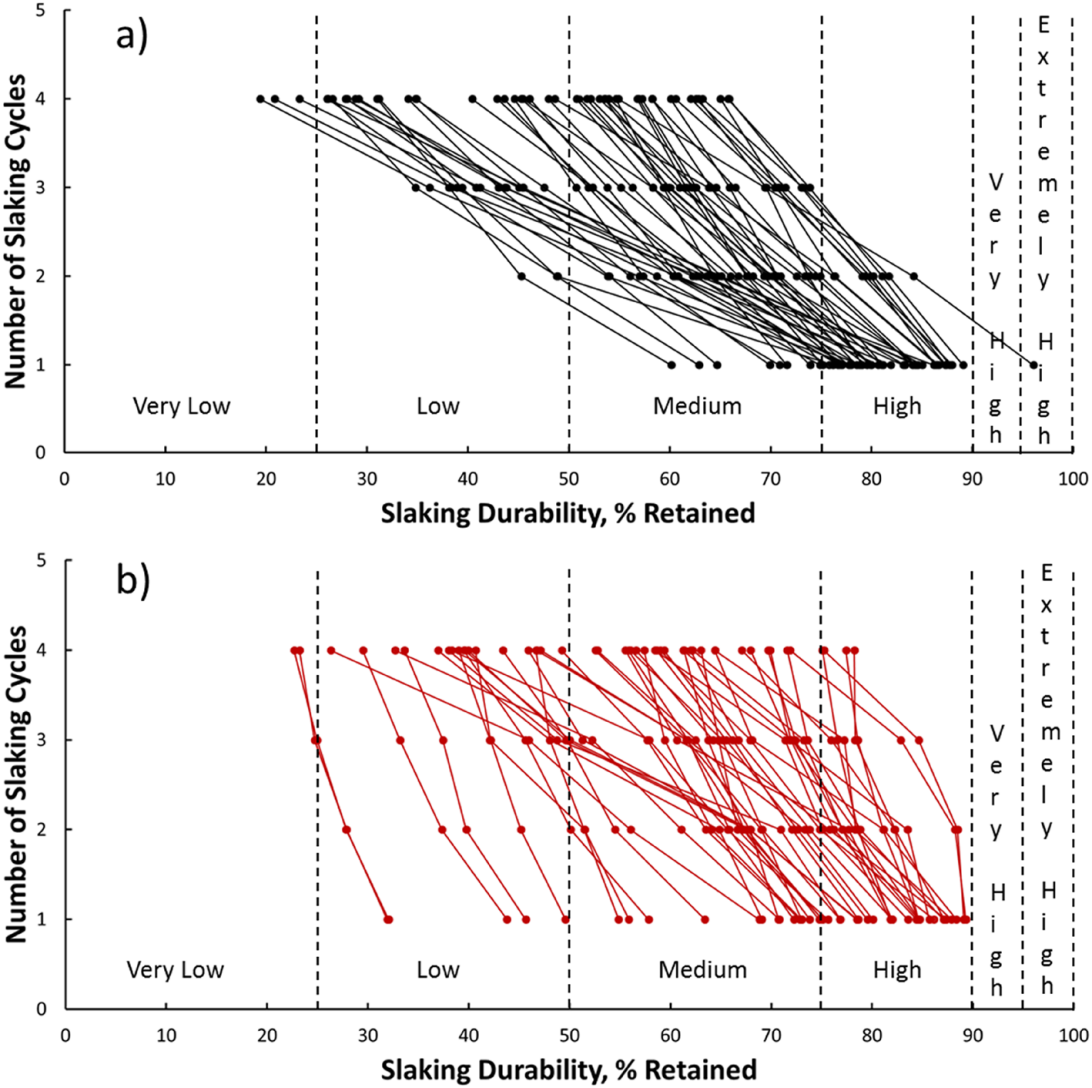
The relationship between number of slaking cycles and slaking durability, % retained for 48 slaking samples and slaked with (**a**) distilled water and (**b**) groundwater including SDI Classification^1^.

**Figure 12:**
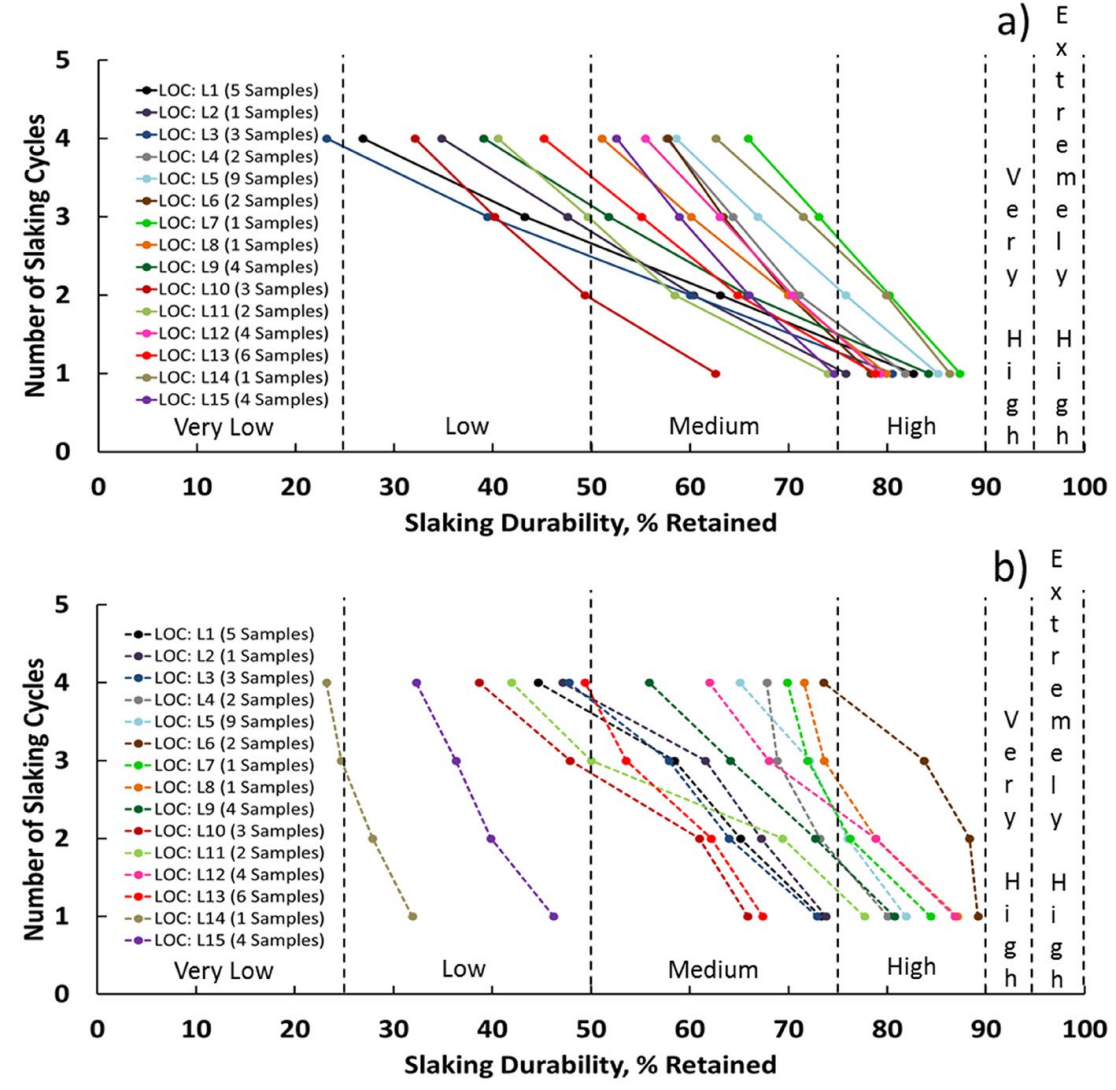
The relationship between number of slaking cycles and slaking durability, % retained for samples collected from 15 locations and slaked with (**a**) distilled water and (**b**) groundwater including SDI Classification^1^.

Table 3 presents the mean SDI classification of the evaporitic rock samples prepared from 48 evaporitic rock blocks for each location from the first to the fourth cycle (I_d1_–I_d4_) with distilled and groundwater as slaking fluids. The results showed that the evaporitic rock durability classification in terms of the second cycle, which is commonly considered and accepted, varied from high to low with either distilled or groundwater as the slaking fluid. However, the highest and lowest durability were perceived in locations 8 and 10 and 6 and 14 after the second cycle with distilled and groundwater as the slaking fluids, respectively. Figures 11a,b and 12a,b illustrate the relationships between the number of the slaking cycles and the slaking durability, %, retained with distilled and groundwater as slaking fluids for 48 SDI tests and 15 locations, respectively. Slaking with groundwater (Fig. 12a,b) was in a wider range compared with that with distilled water (Fig. 11a,b). After four cycles with groundwater and distilled water, the mean weight loss values varied from 11 to 77 wt.% and 4 to 81 wt.%, respectively. These variations in the average slake durability loss from location to location with different slaking fluids could be caused by the quick and effective hydration–dehydration processes, chemical composition of the slaking fluids and mineralogical, chemical and structural nature of the evaporitic rocks. Figures 7, 8, 9, 10 show that the mineralogical composition of the fresh samples (gypsum) differs significantly from that after SDI (bassanite and anhydrite). The mineralogical modification of the investigated samples is combined with differences in textural and chemical composition (Tables 1 and 2).

**Table 3:** Slake Durability Index (SDI) classification for each location using distilled water and groundwater as slaking fluid^1^.

Slaking fluid	Location #	# SDI test samples from rock blocks	Mean I_d1_ (%)	Classification	Mean I_d2_ (%)	Classification	Mean I_d3_ (%)	Classification	Mean I_d4_ (%)	Classification
Distilled water	L1	5	82.64	High	63.03	Medium	43.23	Low	26.81	Low
	L2	1	75.81	Medium to High	60.33	Medium	47.55	Low	34.81	Low
	L3	3	80.49	High	60.03	Medium	39.45	Low	23.13	Very Low
	L4	2	81.80	High	71.06	Medium	64.34	Medium	57.59	Medium
	L5	9	85.13	High	75.78	Medium to High	66.88	Medium	58.58	Medium
	L6	2	78.3	High	70.0	Medium	63.3	Medium	57.8	Medium
	L7	1	87.35	High	80.20	High	73.06	Medium	65.87	Medium
	L8	1	79.91	High	69.94	Medium	60.10	Medium	51.06	Medium
	L9	4	84.17	High	65.79	Medium	51.75	Medium	39.06	Low
	L10	3	62.57	Medium	49.36	Low	40.19	Low	32.09	Low
	L11	2	73.97	Medium	58.45	Medium	49.65	Low	40.51	Low
	L12	4	79.28	High	70.36	Medium	63.02	Medium	55.47	Medium
	L13	6	78.79	High	64.82	Medium	55.06	Medium	45.16	Low
	L14	1	86.33	High	79.88	High	71.49	Medium	62.61	Medium
	L15	4	74.61	Medium	65.98	Medium	58.91	Medium	52.54	Medium
Groundwater	L1	5	73.29	Medium	65.13	Medium	58.45	Medium	44.65	Low
	L2	1	73.81	Medium	67.22	Medium	61.57	Medium	47.13	Low
	L3	3	72.91	Medium	63.97	Medium	57.89	Medium	47.76	Low
	L4	2	80.07	High	73.17	Medium	68.86	Medium	67.80	Medium
	L5	9	81.90	High	75.97	Medium to High	72.14	Medium	65.07	Medium
	L6	2	89.2	High	88.3	High	83.7	High	73.6	Medium
	L7	1	84.40	High	76.22	High	71.95	Medium	69.90	Medium
	L8	1	87.08	High	78.84	High	73.60	Medium	71.58	Medium
	L9	4	80.73	High	72.70	Medium	64.12	Medium	55.91	Medium
	L10	3	65.87	Medium	60.96	Medium	47.88	Low	38.68	Low
	L11	2	77.69	High	69.40	Medium	50.01	Low to Medium	41.96	Low
	L12	4	86.87	High	78.82	High	68.01	Medium	61.98	Medium
	L13	6	67.39	Medium	62.15	Medium	53.56	Medium	49.40	Low
	L14	1	31.91	Low	27.87	Low	24.70	Very Low	23.20	Very Low
	L15	4	46.22	Low	39.85	Low	36.32	Low	32.29	Low
Total rock block samples = 48										

*Groundwater specification: Groundwater level = 10–40 m, Temperature = 21.5–31.2 ($^\circ$C), Salinity = 20,000–65,000 (ppm), pH = 7.14–9.45, TDS = 32,700–175,360 (ppm), EC = 28,100–274000 (μ mhos/cm).

## Conclusions

This work performed SDI tests (one (I_d1_) to four (I_d4_) cycles) with distilled water and groundwater as the slaking fluid on the representative 48 evaporitic rock samples collected from 15 locations in the study area. The findings of this study are as follows:

1. The comprehensive macroscopic and microscopic descriptions of the representative evaporitic rock blocks and the SDI test samples before and after the SDI tests with distilled water and groundwater exposed quite a few variations in their mineralogical compositional and textural structures, which might be directly related to their specific existing environment and the nature of evaporitic rocks.
2. The variations in the mineralogical composition and textural structures of the evaporitic rock samples greatly controlled and affected the SDI test results. Therefore, the SDI test results with the rock sample details of macroscopic and microscopic descriptions must be evaluated.
3. The slaking fluid had a greater influence on the chemical composition of evaporitic rock than mineralogical and textural variations, which depend on the standard of the SDI test.
4. The slaking behavior of evaporitic rocks with groundwater was different compared with that of slaking with distilled water, indicating a wide range. This certainly represented the actual degradability behavior of evaporites in the study area, which was missing and also provided a comparative insight into the degradability behavior of evaporitic rocks with distilled water as a slaking fluid.
5. After four times of cycling of the evaporitic rock samples with distilled water and groundwater, the mean slake durability weight loss was found to be approximately 11–77 and 4–81 wt.%, respectively. Hence, the evaporitic rocks in the study area could be classified with high to low durability.

This comprehensive study could help engineers and decision-makers in building durable, safe and economical structures within the evaporitic rocks in the study area and elsewhere. Nevertheless, note that the results in this study are valid only for the designated rock type. Extending this to other rock types requires more detailed studies.

## Data Availability

The datasets generated and/or analyzed during the current study are available from the corresponding author on reasonable request.
